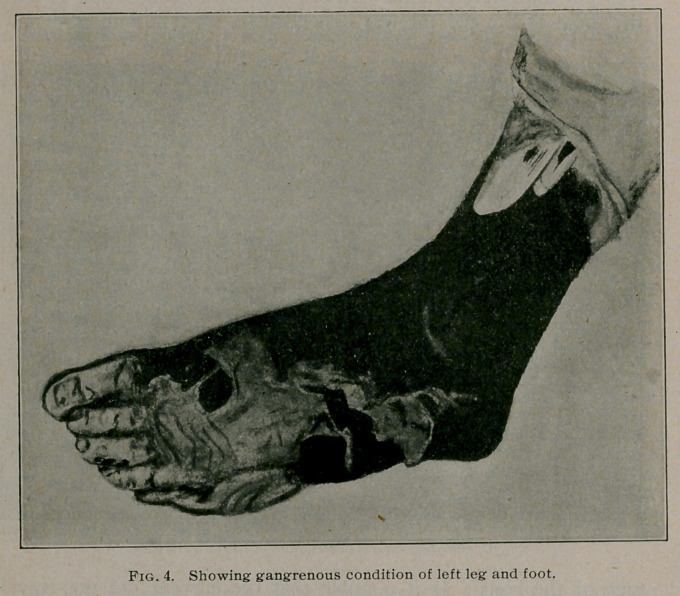# Extensive Gangrene Following Contact with a Live Wire1Read at the thirty-ninth annual meeting of the Medical Association of Central New York, at Syracuse, October 16, 1906.

**Published:** 1907-01

**Authors:** Nathan Jacobson

**Affiliations:** Syracuse, N. Y.; Professor of Surgery, College of Medicine, Syracuse University. Surgeon to St. Joseph’s Hospital


### Extensive Gangrene Following Contact with a Live Wire.[1]

By NATHAN JACOBSON, M. D., Syracuse, N. Y.

Professor of Surgery, College of Medicine, Syracuse University.
Surgeon to St. Joseph's Hospital.

ABOUT six o'clock on the evening of June 15, 1906, F. H. a boy of 12 years of age was brought into St. Joseph's hospital at Syracuse. He was in a state of profound shock. He had a running pulse and complained of extreme pain in his left upper as well as in both lower extremities.

Shortly before that hour he had been on the roof of an abandoned pumphouse in a salt yard hunting for birds' nests. He slipped and began to slide down from the roof. To save himself from falling to the ground a distance of more than twenty feet, he grasped a large electric wire which happened to be one of those furnishing the current to the "White City," a summer resort in the neighborhood of Syracuse. At the time of day when the boy received his injury these wires carry sixty-six hundred volts and have an amperage of 90. The two wires which furnish this service are placed about three feet apart. It is assumed that as he seized one wire with his left hand, his left foot came in contact with the other wire while his right foot still rested on the roof of the pumphouse. He fell to the ground and was picked up in a collapsed condition. I saw him soon after his admission into the hospital.

The left hand was drawn into a state of extreme flexion and rotation and it as well as the lower part of the forearm were quite colorless. On the ulnar side of the elbow there was evidence of a burn and in the axilla of the same side there were two marks, one upon the arm and the other on the chest just as

1. Read at the thirty-ninth annual meeting of the Medical Association of Central New York, at Syracuse, October 16, 1906.

though a wire had come in contact with the body at this point. The left lower extremity was likewise in a contracted condition the foot being drawn downwards. It presented the same bloodless condition as did the left upper extremity. The skin of the palm of the left hand was charred.

The patient was anesthetised but even under deep anesthesia it was impossible to straighten out or indeed make any impression on the contracted muscles. Further examination showed that the soles of both feet were burned. Three days later the circulation in the left forearm and hand as well as that of the corresponding leg and foot still failed to be reestablished. A mummified condition of these extremities was apparent and it was evident that

Fig. 1 Showing left upper extremity.

dry gangrene was developing. After another week sepsis began to manifest itself and instead of dry there was beginning moist gangrene of these parts.

On the 30th of June secondary hemorrhage of the forearm occurred and the house surgeon ligated the brachial artery. I present to you photographs showing the degree of gangrene which had developed by July 1 as well as a water colored picture portraying the exact condition of the left foot and leg at this date. It will be readily seen that it was necessary to amputate the leg above the middle third while the arm had to be removed at a point close to the shoulder in order to find sufficient flap covering.

These operations were performed July 3, 1906, Dr. Coon amputating the leg while I amputated the arm. I submit to you also the chart which indicates the course of his temperature, pulse and respiration.

He was exceedingly nervous after his operation and the dressings, somewhat painful of course, were made with difficulty. For a time there was considerable contraction of the stump of the leg, but this was overcome by the application of proper splints and free motion was ultimately restored to the joint. Some sinuses persisted in both stumps for a little while, due to the presence of small fragments of necrotic bone which were cast off or removed; after which these entirely healed. His recovery

Fig. 2. Showing anterior destruction of tissue of left leg.

though slow is complete. The boy was dismissed from the hospital Oct. 10, 1906.

I present this brief history as well as the patient because I know of no recorded instance of such extensive gangrene following contact with a live electric wire. In a paper presented by Dr. Carlos F. MacDonald to the New York State Medical Society at its annual meeting in 1892 the history of the first seven judicial electrocutions in this state are given in detail. I quote from his paper as to the voltage and amperage of the fatal electric current used in these executions.

"The electromotive pressure as shown by the readings of the voltmeter taken by Prof. L. A. Landy of Columbia College varied from 1458 to 1716 volts while the ammeter showed a variation in current of from two to seven amperes."

Our patient received a charge of electricity enormously greater than that found necessary to destroy life in these judicial executions. Why the boy was not killed outright I cannot say, receiving as he did more than four times as many volts and from fifteen to forty-five times as many amperes of electricity as are used where death by electricity is premeditated. Probably there was but imperfect contact. Another interesting question is why the force of the current was expended on the extremities of one

FIG. 3. Showing lateral destruction of tissue of left leg.

side of the body. Is it possible that the current did not travel through the entire body; that is to say, through the central nervous system?

There are many recorded deaths resulting from contact with wires carrying only the current used for house lighting. This varies in the different cities from 100 to 110 volts with the alternating current, while the amperage is rather uncertain being usually figured as equivalent to one-half ampere per lamp.

The probability is that where death has resulted under these conditions the wires had become crossed, the dosage of electricity received having been therefore much greater than that which usually passes through the wires. It is a matter of common observation to note that where the hand or fingers, for example, have been burned from contact with a live wire that there is a coincident burn upon the sole of the foot; in other words at the points of contact which render the circuit complete.

Our case is not to be regarded as an electric burn. In some way the arterial blood supply was immediately cut off, either because of the contraction of the arteries through vaso-motor stimulation or the blood in the vessels at once coagulated. But, however induced, it is certain that the circulation in both extremities on the left side was suddenly and permanently arrested.

FIG. 4. Showing gangrenous condition of left leg and foot.

After a very thorough search through our literature, I am unable to find a case similar to the one here presented, where a patient surviving such a powerful charge of electricity suffered from gangrene so extensive as to require a double amputation.

430 SOUTH SALINA STREET.

---

Epilation is of great importance in all follicular inflammations of the skin, since it not only removes a host of microbes but also affords access for antiseptics to the deeper parts of the follicle. —*Int. Jour. Surgery.*

## Figures and Tables

**Fig. 1. f1:**
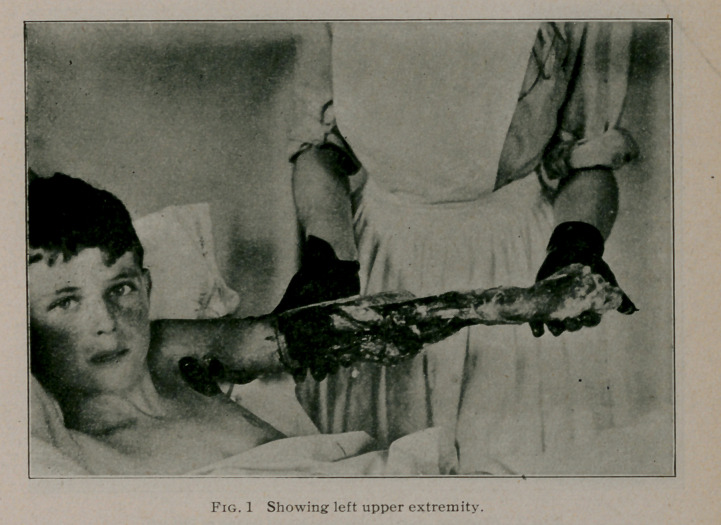


**Fig. 2. f2:**
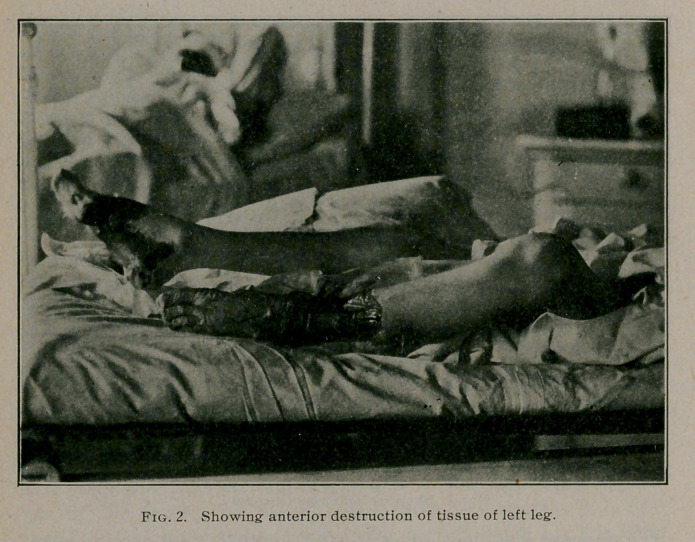


**Fig. 3. f3:**
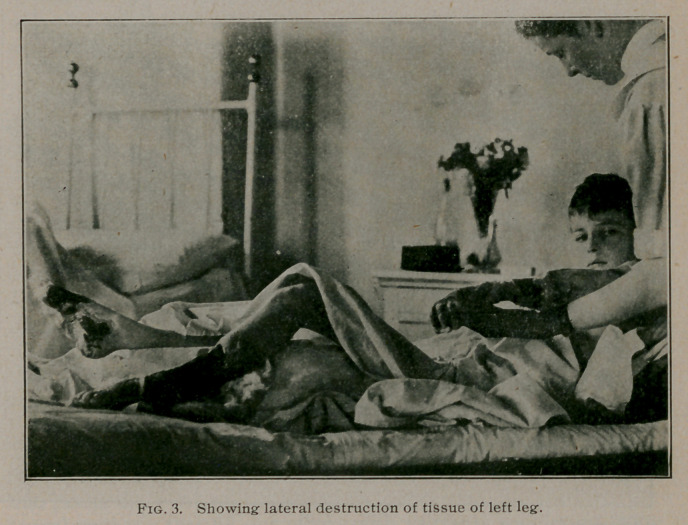


**Fig. 4. f4:**